# Effect of acidic environment on color and translucency of different indirect restorative materials

**DOI:** 10.1186/s12903-024-04218-5

**Published:** 2024-04-19

**Authors:** Abdelaziz A. Omara, Hesham I. Othman, Mohamed F. Aldamaty, Mohamed F. Metwally

**Affiliations:** 1 Department of Fixed Prosthodontics, Faculty of Dental Medicine, Misr University for Science and Technology, Cairo, Egypt; 2 Department of Fixed Prosthodontics, Faculty of Dental Medicine, Al-Azhar University, Cairo, 11651 Egypt

**Keywords:** Color change, Gastric acid, GERD, Hybrid ceramics, Lithium disilicate, Monolithic zirconia, PEEK, Translucency

## Abstract

**Purpose:**

The aim of the current study was to evaluate the effect of simulated gastric acid on the color and translucency of different indirect restorative materials.

**Materials and methods:**

A total of 36 disc-shaped samples were cut by using an isomet saw and divided into four equal groups (*n* = 9) according to the material type: Group Z: translucent zirconia (Ceramill® Zolid ht.^+^ preshade, Amann Girrbach, Koblach, Austria); Group E: lithium disilicate (IPS e.max CAD, Ivoclar Vivadent AG, Schaan, Liechtenstein); Group C: resin nanoceramic (Cerasmart, GC, Tokyo, Japan); Group P: polyether ether ketone (PEEK) (Bettin Zirconia Dentale Italy) veneered with indirect high impact polymer composite (HIPC) (breCAM HIPC, Bredent GmbH & Co. KG, Germany). The samples were immersed in simulated gastric acid (HCl, pH 1.2) for 96 hours at 37 °C in an incubator. The color change (ΔE_00_) and translucency (RTP_00_) were measured every 9.6 hours (one-year clinical simulation) of immersion in simulated gastric acid.

**Results:**

For color change (∆E_00_) and translucency (RTP_00_) among the tested materials, there was a highly statistically significant difference (*P* < 0.001) after every year of follow-up. The color change in both Z and G groups was the lowest after 1 year of acid immersion, followed by that in group H, and the highest change in color was recorded in group P.

**Conclusion:**

High translucent zirconia is recommended in patients who are concerned about esthetic, especially with acidic oral environment.

## Introduction

Currently, ceramic restorations have become the material of choice, especially with the enormous progress in restorative materials, digital dentistry utilities and advancements in technology that have made computer-aided design and computer-aided manufacturing (CAD-CAM) suitable for different clinical situations. Dental ceramics have the properties of biocompatibility, wear resistance, high strength, good color matching and translucency. Since the introduction of monolithic restorations, monolithic zirconia, lithium disilicate glass ceramic, and hybrid ceramics have been used [1, 2].

Full-contour zirconia has high strength, low wear, no veneering chipping, minimal preparation, and long-lasting durability. Although 3 mol% yttria-stabilized tetragonal zirconia polycrystals (3Y-TZP) have the best mechanical properties, their ability to achieve optimal esthetics is still challenged. The yttria content increased in the new generations of CAD-CAM monolithic zirconia systems to 4 and 5 mol% Y-TZP, which have a more isotropic cubic phase, allowing light transmission and, consequently, better translucent monolithic zirconia restorations [3].

Lithium disilicate glass ceramics became the choice for monolithic anterior teeth restorations because they have better mechanical and adequate optical properties than feldspathic porcelains (the best material for esthetics) and have lower strength but greater translucency than conventional zirconia materials. Lithium disilicate can be used to make both anterior and posterior monolithic restorations and additional surface characterizations can be added to tailor opacities and shades [4].

Hybrid ceramics or resin nanoceramics combine ceramic and polymer properties and have similar strength, elasticity, and wear as dentin [5]; however, the color stability of this category is crucial, as clinically observable over time. Therefore, clinicians must be careful when selecting restorative materials because it is one of the most important factors affecting long-term treatment success [6].

Polyether ether ketone (PEEK) is a high-performance polymer that has many applications in engineering and medicine because of its mechanical and chemical properties [7]. PEEK is a suitable material to be used in the dental field because it meets most of the requirements of an ideal material that is used intraorally, including biocompatibility, mechanical strength, temperature resistance, low moisture absorption, low elasticity modulus, flexibility, and chemical wear resistance [8].

The durability of direct and indirect restorations may be affected by multifactorial conditions such as mechanical and chemical factors. Mechanical factors include excessive force due to malocclusion and parafunctional habits such as bruxism and clenching. Chemical factors affect the durability of restorations and include acids that may be generated by extrinsic factors such as fizzy drinks, fruit juices, sports drinks, acidic foods, or medicaments, or intrinsic factors such as an increase in stomach acid due to medical conditions such as gastroesophageal reflux disease (GERD) and bulimia nervosa [9].

Ceramic restorations may be affected by gastrointestinal disorders or continuous vomiting. This may include gastroesophageal reflux disease (GERD), which occurs when the upper esophageal sphincter involuntarily relaxes and allows acid reflux from the stomach to the mouth [9]. Another condition is bulimia nervosa, which is an eating disorder that involves excessive concern about body weight and shape, binge eating, self-induced vomiting or other methods to prevent weight gain [9]. Gastric juice can damage the tooth by demineralizing the enamel, dentin, and cementum. It can also damage ceramic restorations by dissolving their glassy matrix, as it has a very low pH (pH < 1) [10].

Restorative dentistry aims to replace a lost tooth structure with a material that has biological, mechanical, and optical properties that are as close as possible to those of natural teeth. All dental restorations are exposed to complex and varying oral conditions during their service life. Bulimia nervosa and GERD are examples of problems that have unfavorable effects due to the accumulation of acids in the oral cavity causing dental erosion, decreasing the vertical dimension, and dramatically ending with the loss of some teeth [11, 12].

To choose the best aesthetic restorative materials for certain patients, dentists need to know how ceramic or polymeric materials behave when exposed to gastric acid. The literature shows how acidic substances affect the surface of different restorative materials over time and how they influence their color and transparency [13–15]. To our knowledge, the restoration of tooth loss due to chemical action has been described in the literature; however, there is no strong evidence to help clinicians assess the most popular ceramic and polymer restorative materials for aesthetic dental treatment for smile enhancement during long-term exposure to simulated gastric acid without any change in color or translucency.

The objective of the present study was to evaluate the effect of simulated gastric acid on the color and translucency of different indirect restorative materials. Therefore, the null hypotheses were that (a) simulated gastric acid would not affect the color stability of the tested indirect restorative materials along the exposure time; and (b) simulated gastric acid would not affect the translucency of the tested indirect restorative materials along the exposure time.

## Materials and methods

The G-Power statistical power analysis program (version 3.1.9.7) [16] was used for sample size calculation based on the results of Sulaiman et al. [17]. A power analysis was designed to have adequate power to apply a two-sided statistical test to reject the null hypothesis that there is no difference between groups. By adopting an alpha level of (0.05) and a beta of (0.1), i.e., power = 90%, an effect size (d) of (0.684) was calculated based on the results of a previous study. The estimated total sample size was 36 samples (*n* = 9) for detecting differences in color change and translucency parameters between groups.

A total of 36 disc-shaped samples were cut from four indirect restorative materials; Group Z: translucent zirconia (Ceramill® Zolid ht.^+^ preshade, Amann Girrbach, Koblach, Austria); Group E: IPS e.max CAD (Ivoclar Vivadent AG, Schaan, Liechtenstein); Group C: Cerasmart (GC, Tokyo, Japan); and Group P: polyether ether ketone (PEEK) (Bettin Zirconia Dentale Italy) veneered with indirect high impact polymer composite (HIPC) (breCAM HIPC, Bredent GmbH & Co. KG, Germany).

Four restorative materials were used in the current study; two of them were ceramic materials (Zolid ht.^+^ and IPS e.max CAD), one was a shock-absorbable hybrid resin matrix ceramic (Cerasmart), and one was a high-performance polymer represented by PEEK veneered by indirect composite resin to mask the opaque white color of PEEK according to the manufacturer’s recommendation. For the sake of standardization, CAD-CAM blocks/blanks were used, including indirect composite resin, to avoid manual variations as much as possible.

The blocks/blanks were designed into cylindrical shapes by using CAD software (Exocad 3.0 Galway GmbH, Darmstadt, Germany), milled by a 5-axis milling machine (Core Tec touch model 250i, Germany), and then cut with a low-speed diamond saw (Isomet saw 4000, Buehler, Illinois Tool Works Inc., USA) under running water coolant at 4000 rpm to produce disc-shaped samples with final dimensions of 10 **×** 1 mm, except for zirconia, which was larger by 20% to compensate for shrinkage after sintering. A digital caliper (Fisher Scientific Traceable Caliper, USA.) was used to confirm the thickness of the samples after sawing, thus avoiding any optical alterations that could occur due to changes in the thickness of the samples [18]. The diameter of the samples was 10 mm to provide an adequate area for color measurement via a spectrophotometer according to the aperture dimension.

Zirconia samples were sintered in a high-temperature sintering furnace (TABEO-2/M/ZIRKON-100) at a temperature of 1450 °C and a holding time of 1 hour according to the manufacturer’s instructions. IPS e.max CAD samples were crystallized using a Programat P3010 furnace (Programat EP-3010 Furnace, Ivoclar Vivadent) with the specific program for IPS e-max crystallization according to the manufacturer’s instructions (at a temperature of 840 °C and a holding time of 7 min). Cerasmart samples were just finished and polished according to the manufacturer’s instructions. The PEEK samples were veneered with 1.5 mm of HIPC according to the manufacturer’s instructions.

All disc-shaped samples were placed in an ultrasonic bath filled with distilled water for 10 min, removed, dried, and inspected under a magnifying lens for any defects and then polished on the top surface using an EVE Diacera ceramic polishing set (W11DCmf/W11DC, EVE Ernst Vetter GmbH, Pforzheim, Germany) [19].

Finishing and polishing were performed for all the samples because the evidence proving that, compared with those of glazed fired ceramics, the stain resistance and color stability were improved with properly polished surfaces [20–22]. Finishing and polishing were carried out by the same technician for standardization, and the final thickness was verified by using a digital caliper to assure that the thickness was as needed ±0.01 mm.

A generic formula simulating gastric acid was prepared according to previous studies: 0.06 M Hydrochloric acid (HCl) with pH 1.2 [17, 23, 24]. The solution pH was monitored with a pH-meter (AD1030 Adwa 6726 Szeged, Hungary). Each sample was immersed individually with a polished surface facing up in 5 ml of simulated gastric acid for 9.6 hours (576 min) in a 37 °C incubator to represent a one-year clinical simulation and then subjected to testing for color change and translucency. The immersion-measurement cycle, which was repeated every 9.6 hours, was repeated 10 times for each sample until 96 hours immersion time was reached for each sample to represent 10 years of clinical exposure [17, 23, 24].

The samples were measured using a reflective spectrophotometer (Model RM200QC, X-Rite, Neu-Isenburg, Germany) to analyze the optical properties based on ISO/TR 28642 [25]. The aperture size was set to 4 mm and the samples were exactly aligned with the device. The measurements were performed at the center of each sample over a white (CIE L* = 88.81, a* = − 4.98, b* = 6.09) and black backgrounds (CIE L* = 7.61, a* = 0.45, b* = 2.42) relative to the CIE standard illuminant D65 and a 2-degree standard observer and the illuminating/measuring geometry corresponded to CIE 45°/0°. The samples were placed in the center of the measuring port and were kept in the same position for the two backgrounds. The measurements were performed three times for each sample without replacement, and the results were averaged to obtain the single value of a given sample.

The color change (ΔE_00_) of each sample was obtained by calculating the color difference of the sample against a white background (w) before and after acid immersion according to the following eq. [26]:

$$\Delta {\textrm{E}}_{00}={\left[{\left(\frac{\Delta {\textrm{L}}^{\prime }}{{\textrm{K}}_{\textrm{L}}{\textrm{S}}_{\textrm{L}}}\right)}^2+{\left(\frac{\Delta {\textrm{C}}^{\prime }}{{\textrm{K}}_{\textrm{C}}{\textrm{S}}_{\textrm{C}}}\right)}^2+{\left(\frac{\Delta {\textrm{H}}^{\prime }}{{\textrm{K}}_{\textrm{H}}{\textrm{S}}_{\textrm{H}}}\right)}^2+{\textrm{R}}_{\textrm{T}}\ \left(\frac{\Delta {\textrm{C}}^{\prime }}{{\textrm{K}}_{\textrm{C}}{\textrm{S}}_{\textrm{C}}}\right)\ \left(\frac{\Delta {\textrm{H}}^{\prime }}{{\textrm{K}}_{\textrm{H}}{\textrm{S}}_{\textrm{H}}}\right)\right]}^{1/2}$$

To obtain the relative translucency parameter (RTP_00_) of the samples, the CIEDE2000 color difference was calculated from the values obtained from reflectance measurements against white and black backgrounds before and after acid immersion using the following Eq. [27]:

$${\textrm{RTP}}_{00}={\left[{\left(\frac{\Delta {\textrm{L}}^{\prime }}{{\textrm{K}}_{\textrm{L}}{\textrm{S}}_{\textrm{L}}}\right)}^2+{\left(\frac{\Delta {\textrm{C}}^{\prime }}{{\textrm{K}}_{\textrm{C}}{\textrm{S}}_{\textrm{C}}}\right)}^2+{\left(\frac{\Delta {\textrm{H}}^{\prime }}{{\textrm{K}}_{\textrm{H}}{\textrm{S}}_{\textrm{H}}}\right)}^2+{\textrm{R}}_{\textrm{T}}\ \left(\frac{\Delta {\textrm{C}}^{\prime }}{{\textrm{K}}_{\textrm{C}}{\textrm{S}}_{\textrm{C}}}\right)\left(\frac{\Delta {\textrm{H}}^{\prime }}{{\textrm{K}}_{\textrm{H}}{\textrm{S}}_{\textrm{H}}}\right)\right]}^{1/2}$$

where ΔL’, ΔC’, and ΔH’ are the differences in the lightness, chroma, and hue of a given set of samples, respectively. K_L_, K_C_, and K_H_ are parametric factors used to compensate for the mismatch in the experimental conditions; they were fixed at 1 in the current study. S_L_, S_C_, and S_H_ correspond to the weighting functions for lightness, chroma, and hue, respectively. RT represents the rotation function, which is utilized to adjust for the interaction between the differences in chroma and hue in the blue region [27, 28].

To evaluate the ΔE_00_ value, a perceptibility threshold of 50:50% (ΔE_00_ = 0.8) and an acceptability threshold of 50:50% (ΔE_00_ = 1.8) were used according to Paravina et al. [26]. Reliable translucency thresholds were determined by Salas et al. [27] for CIEDE2000 50:50% in which, the translucency perceptible threshold (TPT_00_) = 0.62 units and the translucency acceptable threshold (TAT_00_) = 2.62 units.

The collected data were tabulated and subjected to statistical analysis. Statistical analysis was performed by using one-way ANOVA when comparing more than two groups. A post hoc test was used for multiple comparisons between different variables. Repeated-measures ANOVA was used to compare multiple measures within the same group over time. The Bonferroni correction was used to adjust the *P*-value for multiple comparisons within the same group. The confidence interval was set to 95% and the margin of error accepted was set to 5%. *P*-value ≤0.05 was considered significant, *P*-value < 0.001 was considered highly significant, and *P*-value > 0.05 was considered insignificant.

## Results

### Color change (∆E_00_): Table 1, Fig. 1

For the change in color (∆E_00_) among the tested materials, there was a highly statistically significant difference (*P* < 0.001) after every year of follow-up. Both the Z and E groups showed the same and the lowest color change value after 1 year of acid immersion, followed by group C, and the highest value was recorded for group P. Starting from the evaluation of the second year until the tenth year of evaluation, group Z showed the lowest amount of color change, followed by group E and then group C, and the highest mean value of color change was recorded for group P.

**Table 1:** Comparison of color change (∆E_00_) between groups from 1st to 10th year using ANOVA and Post Hoc tests

Time Intervals	Group Z Mean ± SD	Group E Mean ± SD	Group C Mean ± SD	Group P Mean ± SD	F-test	*P*-value
**1st year**	0.07 ± 0.03 ^Cf^	0.07 ± 0.03 ^Cg^	0.42 ± 0.14 ^Bh^	0.57 ± 0.13 ^Ai^	60.402	< 0.001**
**2nd year**	0.07 ± 0.03 ^Df^	0.42 ± 0.06 ^Ci^	0.55 ± 0.13 ^Bg^	0.78 ± 0.04 ^Ai^	129.634	< 0.001**
**3rd year**	0.07 ± 0.03 ^Df^	0.57 ± 0.13 ^Ch^	1.12 ± 0.08 ^Bf^	2.45 ± 0.09 ^Ag^	1140.334	< 0.001**
**4th year**	0.07 ± 0.03 ^Df^	0.78 ± 0.04 ^Cg^	1.49 ± 0.10 ^Be^	3.90 ± 0.09 ^Af^	4772.525	< 0.001**
**5th year**	0.07 ± 0.05 ^Df^	0.93 ± 0.07 ^Cf^	2.45 ± 0.09 ^Bd^	4.51 ± 0.08 ^Ae^	5806.953	< 0.001**
**6th year**	0.57 ± 0.13 ^De^	1.12 ± 0.08 ^Ce^	3.90 ± 0.09 ^Bc^	4.66 ± 0.07 ^Ad^	4066.535	< 0.001**
**7th year**	0.78 ± 0.04 ^Dd^	1.49 ± 0.10 ^Cd^	4.51 ± 0.08 ^Bb^	6.07 ± 0.06 ^Ac^	9892.402	< 0.001**
**8th year**	0.93 ± 0.07 ^Dc^	2.45 ± 0.09 ^Cc^	4.66 ± 0.07 ^Ba^	6.19 ± 0.07 ^Ab^	8198.000	< 0.001**
**9th year**	1.12 ± 0.08 ^Db^	3.90 ± 0.09 ^Cb^	4.66 ± 0.07 ^Ba^	7.15 ± 0.09 ^Aa^	7761.688	< 0.001**
**10th year**	1.49 ± 0.10 ^Ca^	4.51 ± 0.08 ^Ba^	4.66 ± 0.07 ^Ba^	7.15 ± 0.09 ^Aa^	6353.013	< 0.001**
**RM ANOVA**	41.403	157.096	108.352	112.765		
***P*-value**	< 0.001**	< 0.001**	< 0.001**	< 0.001**		

Different capital letters indicate significant difference at (*P* ≤ 0.05) among means in the same row

Different small letters indicate significant difference at (*P* ≤ 0.05) among means in the same column

*P* > 0.05 is insignificant; **means *P* < 0.001 is highly significant

**Fig. 1:**
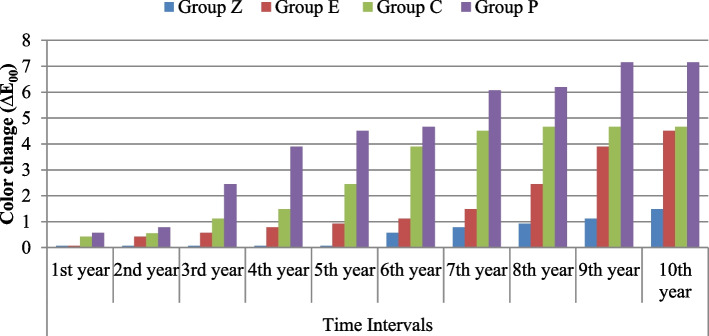
Bar chart illustrating mean values between groups according to color change (∆E_00_) from 1st year to 10th year

Considering the change in color (∆E_00_) within each group, all groups exhibited highly significant differences (*P* < 0.001) between time intervals. The average value for all groups in the 1st year was the lowest, and the highest value was in the 10th year. For group Z, the first significant difference was in the 6th year; for group P, the first significant difference was in the 3rd year; and the first significant difference for groups E and C was in the 2nd year.

### Relative translucency parameters (RTP_00_): Table 2 Fig. 2

The difference in RTP_00_ was highly significant (*P* < 0.001) among the tested materials after every year of follow-up. The lowest changes in RTP_00_ were the same in both Z and E groups after 1 year of acid immersion, followed by group C, and the greatest change was recorded in group P. Starting from the evaluation of the second year until the last year of evaluation, group Z exhibited the lowest change in RTP_00_, followed by group E, then group C, and the greatest change was recorded in group P.

**Table 2:** Comparison of ΔRTP_00_ between groups from 1st to 10th year using ANOVA and Post Hoc tests

**Time Intervals**	**Group Z** **Mean ± SD**	**Group E** **Mean ± SD**	**Group C** **Mean ± SD**	**Group P** **Mean ± SD**	**F-test**	***P*-value**
**Baseline**	48.31 ± 0.02 ^A^	48.31 ± 0.02 ^A^	48.33 ± 0.03 ^A^	48.31 ± 0.02 ^A^	1.414	0.257
**Amount of change**	**MD ± SE**	**MD ± SE**	**MD ± SE**	**MD ± SE**		
**1st Year**	0.02 ± 0.00 ^Cf^	0.02 ± 0.00 ^Cg^	0.49 ± 0.10 ^Bh^	0.72 ± 0.15 ^Ah#^	91.817	< 0.001**
**2nd Year**	0.02 ± 0.00 ^Cf^	0.55 ± 0.06 ^Bh^	0.62 ± 0.06 ^Bg#^	0.98 ± 0.04 ^Ag#^	306.069	< 0.001**
**3rd Year**	0.02 ± 0.00 ^Df^	0.36 ± 0.18 ^Ci^	1.31 ± 0.07 ^Bf#^	2.82 ± 0.13 ^Af﻿§^	266.725	< 0.001**
**4th year**	0.02 ± 0.00 ^Cf^	0.98 ± 0.04 ^Cg#^	1.69 ± 0.12 ^Be#^	4.02 ± 0.14 ^Ae﻿§^	2068.220	< 0.001**
**5th year**	0.03 ± 0.00 ^Df^	1.13 ± 0.08 ^Cf#^	2.84 ± 0.12 ^Bd﻿§^	4.60 ± 0.12 ^Ad﻿§^	2686.183	< 0.001**
**6th year**	0.72 ± 0.15 ^De#^	1.29 ± 0.08 ^Ce#^	4.04 ± 0.13 ^Bc﻿§^	4.80 ± 0.09 ^Ac﻿§^	1885.570	< 0.001**
**7th year**	0.98 ± 0.04 ^Dd#^	1.67 ± 0.13 ^Cd#^	4.62 ± 0.11 ^Bb﻿§^	6.05 ± 0.09 ^Ab﻿§^	3527.573	< 0.001**
**8th year**	1.13 ± 0.08 ^Dc#^	2.82 ± 0.13 ^Cc﻿§^	4.82 ± 0.08 ^Ba﻿§^	6.18 ± 0.11 ^Ab﻿§^	2863.002	< 0.001**
**9th year**	1.29 ± 0.08 ^Db#^	4.02 ± 0.14 ^Cb﻿§^	4.82 ± 0.08 ^Ba﻿§^	7.40 ± 0.10 ^Aa﻿§^	3659.323	< 0.001**
**10th Year**	1.67 ± 0.13 ^Da#^	4.6 ± 0.12 ^Ca﻿§^	4.82 ± 0.08 ^Ba﻿§^	7.40 ± 0.10 ^Aa﻿§^	2851.144	< 0.001**
**RM ANOVA**	33.01	97.90	102.41	184.53		
***P*-value**	< 0.001**	< 0.001**	< 0.001**	< 0.001**		

*MD* Mean Difference, *SE* Standard error, *SD* Standard deviation

Different capital letters indicate significant difference at (*P* ≤ 0.05) among means in the same row

Different small letters indicate significant difference at (*P* ≤ 0.05) among means in the same column

*P*-value > 0.05 is insignificant, **means *P*-value < 0.001 is highly significant

^#^means exceeding TPT_00_ (0.62)

^§^means exceeding TAT_00_ (2.62)

**Fig. 2:**
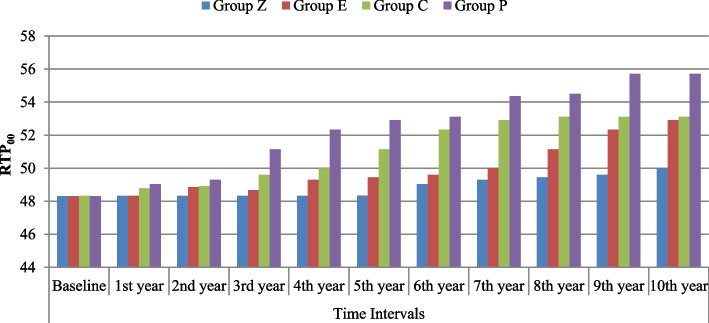
Bar chart illustrating mean values between groups according to RTP_00_ from 1st to 10th year

Considering the RTP_00_ within each group, there was a highly statistically significant difference between time intervals (*P* < 0.001). The lowest change in RTP_00_ for all groups occurred in the 1st year, and the highest change occurred in the 10th year, except for group Z, which experienced no change in RTP_00_ in the first 5 years, while the first significant difference occurred in the 6th year (*P* < 0.001). For group E, the first significant difference occurred in the 2nd year. For groups C and P, the first significant difference was in the 1st year.

## Discussion

Dental restorative materials need to be resistant to chemical damage when used intraorally. Both short-term and long-term exposure to harsh conditions that result from changes in temperature and acidity must be tolerated. Ceramic materials as well as high-performance polymers are options for fixed dental prostheses that need to be evaluated for chemical resistance [29].

In vitro simulation of the effect of gastric acid on the surface of dental restorations depends on the acid concentration, immersion time, and temperature. In the current study, the working pH was 1.2 and the immersion time was 96 h at 37 °C. Each 9.6 hour represents 1 year, so the total duration of immersion was simulated as 10 years of exposure to strong gastric acid [17, 23, 24].

According to previous studies, there is no clear consensus regarding the method of gastric acid simulation or the equivalent duration of replication for an in vivo model. Sulaiman et al. [17] exposed monolithic zirconia to acid solution for 96 hours to simulate the dental structure damage caused by vomiting for more than 10 years. Another study considered gastric acid exposure to CAD-CAM materials for 7.5 h to be similar to 1 month of oral exposure, 45 h to 6 months, and 90 h to 1 year [13], while Backer et al. [30] exposed CAD-CAM materials to simulated gastric acid for 6 and 18 h and concluded that these times represent two and 8 years of exposure of the dental structure to vomiting, respectively.

ISO standard No. 6872 for the solubility test of dental materials [31] uses 4% acetic acid and a 16 h exposure time at 80 °C which is equivalent to 2 years of clinical exposure. However, in the current study a stronger acid (HCl, pH 1.2) was used as an aging solution based on previous studies [17, 23, 24] rather than the ISO standard 6872 to mimic the clinical situation of patients with acid reflux disease. The immersion time was also extended to 96 h at 37 °C, which is supposed to represent more than 10 years of clinical exposure [17, 23, 24].

Various methods are being used in dentistry to evaluate color changes (ΔE) and translucency parameters (TP). The degree of color change or mismatching may be determined visually. However, more accurate reproduction of color, and objective assessments can be obtained through quantification using analytical equipment [32, 33]. In the current study, a spectrophotometer was used to measure the CIE L*a*b* color coordinates on flat ceramic disks. Spectrophotometers are reliable and accurate, which can help dentists choose the right shade, and researchers evaluate the color stability of dental restorations. Furthermore, many previous studies have confirmed the validity of this method [33–35].

The null hypothesis of the present study was rejected, as it was assumed that the simulated gastric acid would not affect the color stability or translucency of the tested indirect restorative materials along the time of exposure.

Zolid ht.^+^ (Group Z) showed a lower ΔE_00_ value below PT (0.8) until the 7th year of acid immersion and within the AT (1.8) until the end of the test. IPS e.max CAD (Group E) showed ΔE_00_ values below PT during the 4 years of immersion and until the end of the 7th year were within AT; then, the last 3 years were unacceptable. Cerasmart (Group C) had a ΔE_00_ below the PT during the first 2 years and within the AT until the end of the 4th year of acid immersion; subsequently the ΔE_00_ became unacceptable starting from the 5th year until the end of the test. The worst scenario was found with PEEK/composite (Group P), as it started to be unacceptable from the 3rd year.

The current study showed evident interactions indicating that the tested restorative materials are not chemically inert but exhibit different signs of degradation in an acidic environment, which agrees with the findings of other previous studies [17, 23, 29]. The results of this study revealed that the change in color was highly significantly different among the tested materials after every year of follow-up. Both the Z and E groups showed the lowest and the same color change values after 1 year of acid immersion, followed by group C, and the highest value was recorded for group P. Starting from the evaluation of the second year until the tenth year of evaluation, group Z showed the lowest amount of color change, followed by group E, and then group C and the highest value of color change was recorded for group P.

This could be attributed to the chelating effect of acid, which can cause degradation, ionic dissolution and the release of alkaline lithium and aluminum ions; these processes are less stable in the glassy phase than in the crystalline phase and result in the dissolution of the ceramic silicate network [17, 29, 36].

The chemical composition, microstructural defects, phase distribution, and crystal size affect the optical properties of zirconia. The purpose of using > 3 mol% yttria stabilized zirconia was to improve the optical characteristics by changing the sintering conditions, and by making the alumina particles smaller, fewer, and in different places in the structure of zirconia [37–39].

Aging factors may affect polymer-based materials more than monolithic ceramics because of polymer infiltration and many polymer-particle interfaces [40]. Resin composites are composed of monomers and inorganic filler particles such as quartz, zirconia, or borosilicate. Chemical erosion of resin can occur due to gastric acid exposure, which manifests as soft resin, protruding filler particles, voids, and cracks regarding the time of exposure [41].

In the current study, the translucency was almost the same for all materials at the baseline of the test, but after acid immersion, it was significantly different (P<0.001) for each material. Zolid ht.^+^ had the lowest change in RTP_00_, followed by IPS e.max CAD, Cerasmart, and finally the PEEK/composite group with the highest change. This could be due to the differences among the RTP_00_ values of the materials, which depend on the chemical composition, crystalline content, grain size and microstructural variations.

Zolid ht.^+^ (Group Z) exhibited a lower ΔRTP_00_ value below TPT_00_ (< 0.62) until the 5th year of acid immersion and within the TAT_00_ (2.62) until the end of the test. IPS e.max CAD (Group E) showed ΔRTP_00_ values below TPT_00_ during the first 3 years of immersion and until the end of the 7th year were within TAT_00_; then, the last 3 years were unacceptable. Cerasmart (Group C) had a ΔRTP_00_ below the TPT_00_ during the first 2 years and within the TAT_00_ until the end of the 4th year of acid immersion; subsequently the ΔRTP_00_ became unacceptable starting from the 5th year until the end of the test. Finally, for PEEK/composite (Group P), it started to be unacceptable from the 3rd year.

The translucency results of the current study were in agreement with those of Sulaiman et al. [17] but were in disagreement with those of Kulkarni et al. [14], who did not find any significant effect of gastric acid immersion on dental ceramics (feldspathic porcelain, IPS e.max CAD, and monolithic zirconia). This may be due to the use of different methodologies, pH values, and immersion times, as the researchers dipped samples in gastric acid (pH 2) for 2 minutes and then rinsed them with deionized water for 2 minutes; moreover, the procedure was repeated 6 times a day for 9 days.

The current study did not test the materials against different pH values, which could be a limitation. Exposure to different pH values could have provided additional insight into the optical properties of the tested restorative materials. Additionally, the effect of acidic media on the flexural strength should be further investigated.

## Conclusion

Within the limitations of this study, the following conclusions may be drawn:

1. The material type is a crucial factor in determining whether the color change caused by gastric acidity will be perceivable to the human eye and clinically unacceptable or not.
2. High translucent zirconia restorations are recommended for patients who are concerned about esthetics, especially with acidic oral environment.
3. In an acidic oral environment, lithium disilicate, hybrid ceramic, and PEEK veneered with composite resin, are not recommended for aesthetic rehabilitation in patients suffering from conditions such as GERD or bulimia nervosa.

## Data Availability

The dataset used and analyzed data are available from the corresponding author upon reasonable request.
